# First case of childhood Takayasu arteritis with renal artery aneurysms

**DOI:** 10.1186/1546-0096-8-21

**Published:** 2010-07-24

**Authors:** Tahar Gargah, Mouna Ben Harrath, Haythem Bachrouche, Hatem Rajhi, Taeb Ben Abdallah, Mohamed R Lakhoua

**Affiliations:** 1 Department of Pediatric Nephrology, Charles Nicolle Hospital, Tunis, Tunisia; 2 Department of Radiology, Charles Nicolle Hospital, Tunis, Tunisia; 3 Department of Nephrology, Charles Nicolle Hospital, Tunis, Tunisia

## Abstract

Takayasu arteritis is a large vessel systemic granulomatous vasculitis characterized by stenosis or obliteration of large and medium sized arteries. It commonly involves elastic arteries such as the aorta and its main branches. Renal artery involvement is rare and has not been reported in a child. We report a 12-year-old boy with Takayasu arteritis who developed severe hypertension, proteinuria, microscopic hematuria and renal dysfunction. Conventional angiography demonstrated aneurysms of both renal arteries and multiple microaneurysms of the superior mesenteric artery. This case report illustrates that the children with Takayasu arteritis can develop renal involvement resulting in hematuria, proteinuria and even renal failure.

## Background

Takayasu Arteritis (TA) is a chronic, idiopathic and granulomatous vasculitis of the large arteries. It involves primarily the aorta, especially aortic proximal branches, and occasionally the pulmonary arteries. This inflammatory condition may result in stenosis, occlusion, dilatation or aneurysm of the involved arteries [1].

The disease is widely prevalent in Asian populations. It is the most frequent cause of renovascular hypertension in India [2]. The peak incidence is in the third and fourth decades of life but the onset of illness may occur earlier, including in childhood [3]. The etiology and predisposing factors of TA are as yet unknown. A history of tuberculosis has been associated with TA, an autoimmune basis for the disease appears likely, and genetic and environmental factors may also play important etiologic roles [4].

The clinical presentation is very heterogeneous and involves two stages: an initial inflammatory process or "prepulseless phase" with variable systemic manifestations occurring, followed by a later "pulseless phase" with multiple arterial occlusions and stenosis producing symptoms of cerebral, visceral, or extremity ischemia [5].

We report the case of a 12-year-old boy with a history of peripheral tuberculous lymphadenitis. He developed severe hypertension, proteinuria, microscopic hematuria and renal failure. Clinical and laboratory investigations suggested TA. Conventional angiography revealed several vascular abnormalities related to his TA disease, including renal artery aneurysms.

## Case report

A 12 years old Arab boy presented to our hospital with the chief complaints of headache and abdominal pain. He had a history of cervical tuberculous lymphadenitis or scrofula at the age of nine and a uveitis episode two years ago. He had no chest or back pain, no history of ischemic episodes and no limb claudication. There was no family history of hypertension or renal disease.

On admission, he was afebrile. His heart rate was 110 beats per minute. His right and left brachial blood pressures were respectively 170/110 and 160/100 mmHg. He was found to have a systolic bruit in the left external carotid artery. Peripheral pulses were full and no bruit was detected over the abdomen or elsewhere. On the skin examination, multiple erythema nodosum lesions were noted., His neurological exam was normal. An eye examination revealed normal visual acuity. He had a clear cornea and normal anterior chambers.

Laboratory investigations showed following results: A urinalysis revealed microscopic hematuria and proteinuria. A 24 hour urine revealed urinary protein loss of 25 mg/kg/24 hours. Blood urea was elevated at 12 mmol/l with a serum creatinine of 156 μmol/l. The Westergren erythrocyte sedimentation rate was very elevated at 120 mm/hour. The C-reactive protein was 7.8 mg/dl. A protein electropheresis revealed elevated alpha and gamma globulins. without signs of nephrotic syndrome. His serum was negative for rheumatoid factor, antinuclear factors and antineutrophil cytoplasmic antibodies. The complement factors C3 and C4 were normal. The hepatitis B antigen was negative. The tuberculin skin test was positive.

Chest radiography was normal. No coronary blood vessel abnormalities were found by echocardiography. Renal Doppler ultrasound scanning was interpreted as normal.

Cervical Doppler ultrasonography showed a narrowing in both carotids with thick-walled, small caliber carotids (carotid wall thickness: 4 mm). There was a moderate accelelerated flow through the carotids (150 cm/s).

**Figure 1:**
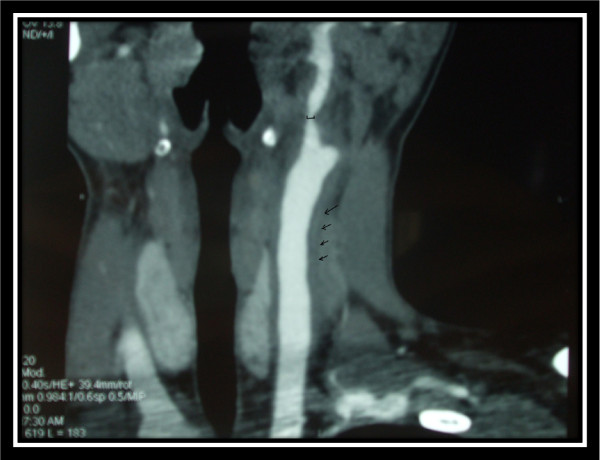
**Cervical CT angiography shows regular thickening of the left common carotid artery and left external carotid stenosis**.

**Figure 2:**
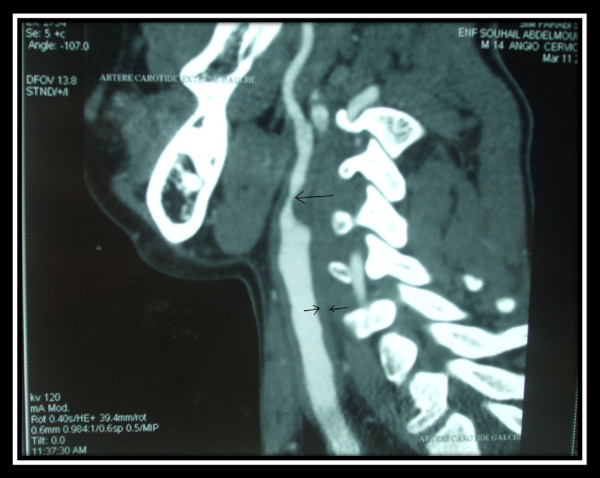
**Cervical CT angiography shows irregular thickening of the left common carotid artery wall**.

**Figure 3:**
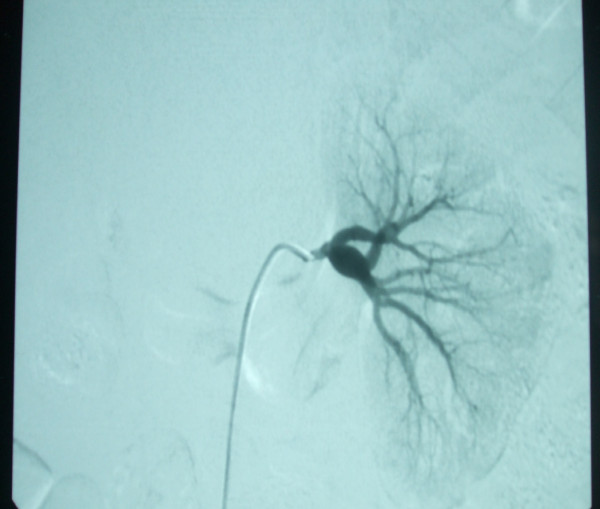
**Conventional angiography showed a renal artery aneurysm**.

**Figure 4:**
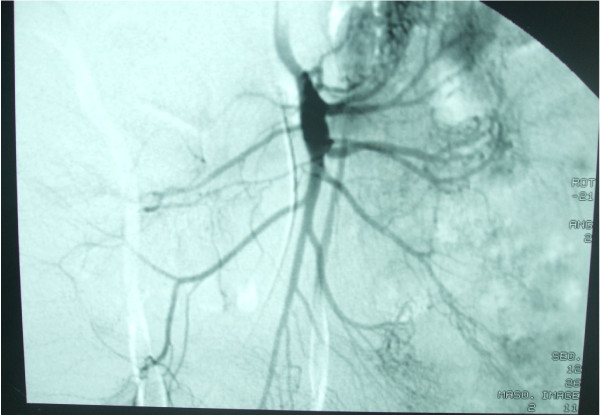
**Conventional angiography showed aneurysm in the origin of superior mesenteric artery**.

Thoracic CT angiography showed regular thickening of the left common carotid artery. There was a marked stenosis (50%-60%) at the origin of the left external carotid artery (Figures 1 and 2). Furthermore, conventional angiography revealed an aneurysm in both renal arteries and an aneurysm in the origin of superior mesenteric artery (Figures 3 and 4).

The patient was treated with prednisolone with an initial dose of 2 mg/Kg/day. This dose was maintained for 30 days and then a slow taper of the prednisolone was started.

The hypertension was easily controlled by calcium channel blockers (nifedipine). An antiplatelet agent was prescribed to prevent thrombotic complications. At 3 months after initiation of treatment, the patient remained normotensive. The laboratory tests then revealed normal acute phase reactants, the normalization of renal function, and the disappearance of an abnormal urinary sediment.

## Discussion

TA is a rare vasculitis involving only large arteries, with an estimated incidence of 2.6 cases per million persons per year in North America [6].

Clinical manifestations vary greatly depending on the affected arteries, making a early diagnosis difficult [7]. However, it is important to make a correct diagnosis in TA patients as soon as possible. Early diagnosis allows early and appropriate treatment to prevent vascular and visceral complications related to the disease. In our patient, clinical findings, a history of tuberculosis, and angiographic findings suggested the diagnosis of TA.

This patient met the American College of Rheumatology criteria [8] which were primarily designed for adult TA detection [9,10]. Four criteria are needed: age less than 40 years, blood pressure difference > 10 mm Hg between upper extremity and lower extremity pressures, a bruit over subclavian arteries, and angiographic abnormalities. He also fulfills the revised EULAR/PReS criteria for TA [9].

Aneurysm disease in children resulting from TA is unusual [10]. These aneurysms usually affect the aorta or its major branches. The study of Matsumura [11] revealed that in patients with TA, there was a moderately high incidence of aneurysms (31.9%) at various arterial sites in adults. The most common site of aneurysms was the ascending aorta. In her study of Indian patients, Muranjan [10] reported the prevalence of aneurysms mainly in the aortic area of the thoracic aorta to the abdominal aorta. In TA patients in general, typical angiographic findings include a combination of aneurysm formation and cylindrical segmental stenosis or occlusion [12]. Pathologic examination of affected arteries is characterized by a medial degeneration with disruption of the elastic lamina. Giant cells infiltration may be present [13].

TA presents therapeutic challenges especially in children. For our patient, we have used oral corticosteroids as first-line as usually done in adults with TA. Due to what we thought to be an unusual severity of clinical inflammatory and biological signs, we employed a high dose of corticosteroids and kept the dose high for a month before starting a slow 2 year decrease of the dose.

In cases of steroid-resistance or serious side effects related to steroid therapy, other therapies have been proposed. These treatments include oral or intravenous cyclophosphamide, methotrexate and mycophenolate mofetil [14]. More recently, the biologic, anti-tumor necrosis factor agent infliximab have been proposed as a second line treatment [15,16].

Surgery is recommended when patient is in clinical remission of disease to minimize operative risk and avoid complications due to inflammation such as restenosis, anastomotic failure, thrombosis, hemorrhage and infection [17]. The indications for surgery include hypertension with critical renal artery stenosis, extremity claudication limiting activities of daily living, cerebrovascular ischemia or critical stenosis of three or more cerebral vessels, moderate aortic regurgitation, and cardiac ischemia [6]. Our patient so far has had no indication for surgery. The renal function was restored, the hypertension was controlled, and urinary abnormalities disappeared. Patients with TA must be carefully monitored clinically, with laboratory and radiologic tests, and biologically. Currently the radiological monitoring can be provided through non-invasive procedures such as ultrasound and magnetic resonance angiography [18].

## Conclusion

Few cases of TA in children have been reported in the medical literature. The involvement of the renal artery due to TA has not been previously described in children. The diagnosis of this complication is challenging in children with TA, but with a higher index of suspicion for involvement of the renal arteries as well the more classically involved vessels and with the appropriate clinical, laboratory, and radiological evaluation, this potentially serious renal disease in children with TA may be diagnosed and treated without ensuing renal damage.

## Competing interests

The authors declare that they have no competing interests.

## Authors' contributions

All authors contributed to the analysis and interpretation of the data. TG, MBH and HB wrote the initial manuscript draft, based on guidance from MRL. HR and TBA performed the analysis and interpretation of radiological data. All authors critically reviewed and revised drafts. All authors read and approved the final manuscript.
